# Clinical experience with two frameless stereotactic radiosurgery (fSRS) systems using optical surface imaging for motion monitoring

**DOI:** 10.1120/jacmp.v16i4.5416

**Published:** 2015-07-08

**Authors:** Guang Li, Ase Ballangrud, Maria Chan, Ruimei Ma, Kathryn Beal, Yoshiya Yamada, Timothy Chan, James Lee, Preeti Parhar, James Mechalakos, Margie Hunt

**Affiliations:** ^1^ Department of Medical Physics Memorial Sloan Kettering Cancer Center New York NY USA; ^2^ Department of Radiation Oncology Memorial Sloan Kettering Cancer Center New York NY USA

**Keywords:** brain cancer, stereotactic radiosurgery, image‐guided radiotherapy, optical surface imaging, real‐time motion monitoring

## Abstract

The purpose of this study was to compare two clinical immobilization systems for intracranial frameless stereotactic radiosurgery (fSRS) under the same clinical procedure using cone‐beam computed tomography (CBCT) for setup and video‐based optical surface imaging (OSI) for initial head alignment and intrafractional motion monitoring. A previously established fSRS procedure was applied using two intracranial immobilization systems: PinPoint system (head mold and mouthpiece) and Freedom system (head mold and open face mask). The CBCT was used for patient setup with four degrees of freedom (4DOF), while OSI was used for 6DOF alignment prior to CBCT, post‐CBCT setup verification at all treatment couch angles (zero and nonzero), and intrafractional motion monitoring. Quantitative comparison of the two systems includes residual head rotation, head restriction capacity, and patient setup time in 25 patients (29 lesions) using PinPoint and 8 patients (29 fractions) using Freedom. The maximum possible motion was assessed in nine volunteers with deliberate, forced movement in Freedom system. A consensus‐based comparison of patient comfort level and clinical ease of use is reported. Using OSI‐guided corrections, the maximum residual rotations in all directions were 1.1°±0.5° for PinPoint and 0.6°±0.3° for Freedom. The time spent performing rotation corrections was 5.0±4.1 min by moving the patient with PinPoint and 2.7±1.0 min by adjusting Freedom couch extension. After CBCT, the OSI–CBCT discrepancy due to different anatomic landmarks for alignment was 2.4±1.3 mm using PinPoint and 1.5±0.7 mm using Freedom. Similar results were obtained for setup verification at couch angles (<1.5 mm) and for motion restriction: 0.4±0.3 mm/0.2°±0.2° in PinPoint and 0.6±0.3 mm/0.3°±0.1° in Freedom. The maximum range of forced head motion was 2.2±1.0 mm using Freedom. Both intracranial fSRS immobilization systems can restrict head motion within 1.5 mm during treatment as monitored by OSI. Setting a motion threshold for beam‐hold ensures that head motion is constrained within the treatment margin during beam‐on periods. The capability of 6D setup is useful to improve treatment accuracy. Patient comfort and clinical workflow should play a substantial role in system selection, and Freedom system outperforms PinPoint system in these two aspects.

PACS number: 87.53.Ly, 87.55.D‐, 87.57.Q‐, 87.6s.L‐, 87.85.gi

## I. INTRODUCTION

For small brain tumor, including malignant metastatic lesion and benign primary tumor, the single‐fraction stereotactic radiosurgery (SRS) is an effective treatment modality. Conventional frame‐based SRS is an invasive procedure to immobilize patient, localize the lesion, and provides high local control with acceptable toxicity. SRS is especially useful for nonoperable, deep seated, and disperse multiple lesions. SRS planning employs multiple beams or arcs, coupled with a highly conformal dose distribution and a sharp dose falloff outside of the target.^(1)^ An ablative radiation dose will be delivered to the lesion in a single fraction. Therefore, it requires a high level of accuracy and precision during the initial target localization and treatment delivery. Frame‐based SRS localization and immobilization systems achieve these objectives by invasively fixing the patient's head to a metal frame, referencing the lesion to the frame system, and locking the head frame to the treatment couch.

With recent advancements in image‐guided patient setup and motion monitoring, SRS has gradually started to transform from a frame‐based to frameless SRS (fSRS).^2^, ^3^, ^4^, ^5^, ^6^, ^7^, ^8^ Three‐dimensional (3D) image guidance, such as cone‐beam computed tomography (CBCT), has become a standard for initial target localization, and positioning within submillimeter accuracy is then combined with a modestly stringent immobilization system and intratreatment head motion surveillance. Early approaches to intratreatment motion monitoring involved the application of an array of six infrared reflective markers mounted on a tray held by the patient via a bite‐block.^9^, ^10^ The speed of motion tracking was up to 30 frames per second (fps) with six degrees of freedom (6DOF), provided that the bite‐block maintained the rigid relationship between the tray and the head. More recently, intratreatment motion monitoring has shifted toward markerless approaches using video‐based optical surface imaging (OSI), reducing the need for patient compliance and simplifying fSRS treatment.^11^, ^12^, ^13^ With OSI system, a region of interest (ROI) on the 3D facial surface is used to align the reference and treatment images. The surface images may utilize $∼n\times 10^{2}-10^{3}$ points to track with a frame rate of 2–5 fps. This tracking rate is sufficient to detect head movement within a modestly stringent immobilization device and trigger a beam‐hold when the motion exceeds a predefined threshold.

AlignRT (VisionRT, London, UK) is an OSI system that has been used at our institution and others to assist in initial patient setup and motion monitoring for fSRS procedures.^11^, ^12^ Several immobilization systems for fSRS have been described, including one that initially utilized a Mayo mold only,^(11)^ but later evolved into a system with a simpler head cushion and an open face mask.^(13)^ This system provides moderate head motion restriction and ensures target position during treatment using AlignRT motion monitoring. Another reported clinical fSRS system uses a head mold and a more restrictive mouthpiece that includes a personalized dental mold and reinforced vacuum‐suction between the dental mold and the upper hard palate.^(12)^ This system provides a high degree of motion restriction, thereby minimizing the possibility of any large sudden movement, but patient comfort and clinical workflow may be affected. The use of 6DOF correction could help improve the accuracy of the image‐guided fSRS setup.^(14)^

In this study, we evaluate an immobilization system that combines a Mayo mold with an open face mask that provides moderate head restriction while retaining patient comfort. We hypothesized that this system would restrict head motion at least as well as the systems that include either the head mold alone^(11)^ or an open face mask with a simple head cushion.^(13)^ The system described is the Freedom system (CDR Systems, Calgary, AB, Canada), which comes with a couch extension board for the adjustment of head rotation. Using the same fSRS treatment protocol, we compare the Freedom system with the PinPoint bite‐block system (Aktina Medical, Congers, NY), which we developed and used in our clinic.^(12)^ Several parameters of the fSRS treatments were quantified and compared, including residual head rotation, motion amplitude, patient comfort, and workflow.

## II. MATERIALS AND METHODS

### A. General frameless SRS procedure

**Figure 1:**
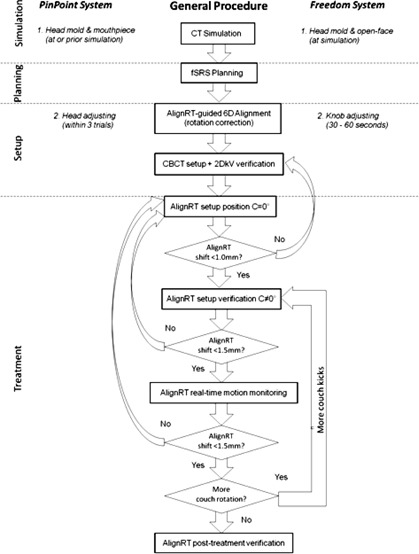
Clinical workflow of frameless SRS (fSRS) using OSI to monitor motion. Two different patient immobilization systems are applied. Note: Aktina PinPoint rotation‐adjusting board was not used due to the rotation center distanced from the head, while CDR Freedom rotation‐adjusting couch extension (head support) was utilized.

The general fSRS procedure and workflow are shown in Fig. 1. At simulation, patients were positioned and scanned with computed tomography (CT) using a neutral head position, which facilitates real‐time acquisition of high‐quality surface images of the ROI from ceiling‐mounted AlignRT cameras (VisionRT, London, UK) in a treatment room. Three ball bearings (BBs,φ=1 mm) were placed on the head surface of the patient in superior axial plane to mark an internal reference point that defined the treatment isocenter. The fSRS plan subsequently developed usually consisted of nine to twelve conformal beams designed using the iPlan system (BrainLAB AG, Feldkirchen, Germany). Typically 18–21 Gy to the 80% isodose line was prescribed for metastatic lesions with the dose distribution normalized to the isocenter.

The preparation for the OSI‐based initial alignment and motion monitoring was done prior to treatment. The ROI was defined from the external contours of the simulation CT images transferred from the planning system to AlignRT via DICOM RT (Digital Image Communication in Medicine for Radiotherapy). This ROI generally included the anterior facial area that extended from the philtrum superiorly to the forehead and laterally cheek‐to‐cheek. When a new AlignRT reference image was acquired and used, the ROI was automatically mapped from the DICOM image to the new reference image. Visual verification of surface registration was performed.

At treatment, the patient was first setup conventionally using room lasers with shifts from the triangulation BBs. Next, using AlignRT guidance with 6DOF, static or real‐time delta (RTD) mode, residual head rotation was corrected by adjusting either the patient when using PinPoint system or a rotation‐adjustable couch extension when using the Freedom system. Efforts were made to correct the all translational and rotational shifts indicated by AlignRT prior to moving to the next step, which consisted of cone‐beam CT (CBCT)‐guided setup. During CBCT acquisition, registration of the CBCT and planning CT and subsequent physician approval, the AlignRT RTD monitoring was used to ensure that the patient did not move.^(12)^ After the patient position was finalized, based on the CBCT and confirmed using two‐dimensional kilovoltage (2D kV) radiographic imaging with bony landmark alignment, a final static AlignRT image (${\text{AlignRT}}_{\text{postCBCT}}$) was captured for use during intratreatment motion monitoring. The residual AlignRT shifts at that time were used to quantify the residual discrepancies between the AlignRT and CBCT setups.

As treatment progressed and the couch was rotated at each successive treatment couch angle, an additional AlignRT static image (${\text{AlignRT}}_{\text{couch}}$) was captured. The residual discrepancies between each ${\text{AlignRT}}_{\text{couch}}$ image and ${\text{AlignRT}}_{\text{postCBCT}}$ were used to verify the current patient positioning. The tolerance for the residual discrepancies between these AlignRT images was 1.5 mm and 1° in any direction. If the tolerance was exceeded, the action was to rotate the couch back to zero, capture another AlignRT image, and compare it to AlignRT_postCBCT_. Discrepancies of more than 1.0 mm were assumed to be indicative of true patient motion that then led to a new CBCT imaging of the patient. The tolerance of 1 mm at zero couch angle is more restrictive than that at nonzero couch angles, to allow for the additional uncertainty from couch walk and the angular dependency of the AlignRT system. After setup verification at each couch angle, a new AlignRT reference image was acquired, the previously defined ROI was automatically mapped to the new reference image, and the patient position was then monitored using the RTD feature with a threshold of 1 mm. Motion outside of the 1 mm threshold led to a manual or automatic beam‐hold until the RTD signal fell back within the threshold. At the end of treatment, a final static AlignRT image was captured for comparison with the initial post‐CBCT image, AlignRT_postCBCT_.

### B. Immobilization systems

#### B.1 Aktina PinPoint

**Figure 2:**
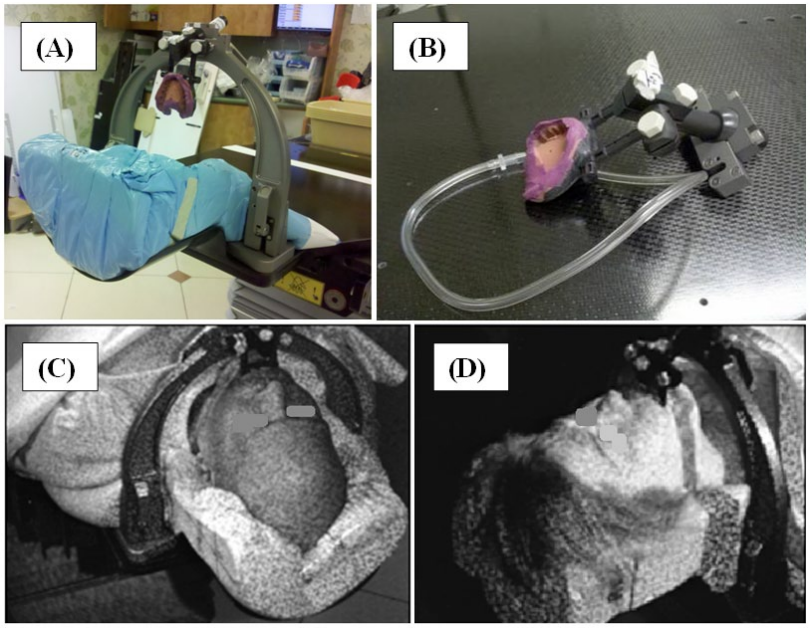
Aktina PinPoint immobilization system (a) and vacuum‐suction customized mouthpiece (b). Two patients immobilized with the system (c) and (d).

Aktina PinPoint system contains a head mold and a mouth piece, which “lock” the head in six translational directions: the head mold prevents motions in the posterior, superior, left and right directions, while the mouthpiece restricts anterior and inferior motion. The mouthpiece includes a customized dental mold and a vacuum suction between the mold and the upper hard palate. Mouthpieces were made by a dentist prior to CT simulation. The PinPoint system is shown in Fig. 2. It must be noted that PinPoint rotation adjuster was not utilized as its rotation center was distant from the intracranial structure. Patients were tattooed at the three BB positions near the eyebrow and lateral hairline.

During setup, the patient was first aligned using the triangulation tattoos while the head was locked in place with the mouthpiece. The entire anterior–superior facial region from the forehead hairline to the philtrum was used as the ROI. Using AlignRT 6DOF alignment, head rotation was assessed. Rotations exceeding 1.0° in any direction were corrected manually by unlocking, adjusting, and relocking the position of the patient, followed by reassessment using static AlignRT imaging. AlignRT RTD mode was not used during this procedure as continuous RTD monitoring provided little guidance for the discrete head adjusting process. In order to minimize patient discomfort, no more than three attempts to manually correct the head rotation were conducted per patient. Once the initial head position of the patient was set in the PinPoint system, the aforementioned general fSRS procedure was applied.

Twenty‐five patients with twenty‐nine lesions were treated with single‐faction fSRS using the PinPoint system. All treatments were performed with a Trilogy (Varian Medical, Palo Alto, CA) accelerator and a regular AlignRT three‐camera system.

#### B.2 CDR Freedom

**Figure 3:**
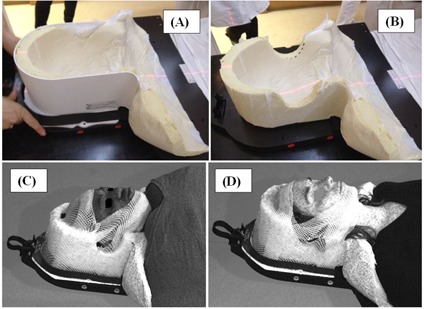
Construction of the Mayo head mold (a) and (b) with open face mask on two volunteers (c) and (d) using the Freedom immobilization system.

The Freedom system consists of a head mold and an open face mask to immobilize the head in all six translational and rotational directions. Compared to the PinPoint system, the open face mask replaced the mouthpiece, thus restricting anterior and inferior motions. The head mold was made first and after the foam hardened, the open face mask was made using a precut thermoplastic material. As shown in Fig. 3, the head, neck, and shoulder were supported. After CT scan, the tumor location was identified relative to the origin based on the three BBs' position and cast lines were drawn on the mask for initial head alignment at setup. The patients were not tattooed.

At treatment, the patient was initially positioned by matching the cast lines with room lasers on the couch extension board, which allows pitch and roll adjustments (<3°). The anterior–superior facial region, excluding the hair in the open portion of the mask, was the ROI (Fig. 2). The rotational axes of the couch extension board for pitch and roll were at the hinge and lateral center of the extension, respectively. The head position was manually adjusted with the mask off to minimize yaw, roll, and pitch rotations under AlignRT RTD guidance. The mask was then placed and locked, which tended to move the head slightly. The new residual rotations were then corrected using the Freedom rotational adjustments and standard couch rotation. The rest of procedure was as described above (General frameless SRS procedure).

Since the CDR Freedom may not provide a head fixation as rigid as the PinPoint system, we did a thorough testing of the Freedom system on volunteers before use on patients. To assess the worst‐case scenario, we tested the maximum motion range on nine volunteers with deliberately forced movements. The volunteers were asked to move right and left and to move their chin up and down. For each move, they held the position for a few seconds and released the force.

Eight patients participated in the clinical evaluation for a total of 29 fractions of either single‐fraction fSRS or hypofraction fSRT treatments. For hypofraction fSRT cases, treatments were performed using single‐fraction criteria aforementioned. At least one SRS physicist was present for the treatments.

### C. Comparison between the two immobilization systems

Several aspects of each immobilization system were characterized with AlignRT imaging, including i) head rotation correction in initial setup, ii) setup verification at different couch angles, iii) possible head motion ranges, and iv) time required for setup and treatment delivery. Data in the above four categories for each system were statistically analyzed and compared. For each system, patient comfort and ease‐of‐use was evaluated by a survey among six physicists and nine therapists who were involved in the use of both systems for simulation and/or treatment. The systems were ranked from 1 to 10 (worst to best) by each survey participants, and the average scores represented the consensus of the users.

## III. RESULTS

### A. Deliberate forced moves in Freedom system

**Figure 4:**
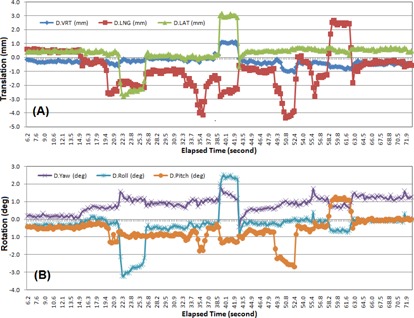
An example of forced motion observed using the Freedom immobilization system. The average results are shown in Table 2. Because of head rotation effects (b), the translational motions (a) at the surface of the patient do not necessarily imply tumor motion, which should be estimated by combining all 6DOF shifts and the location of the tumor (the isocenter is the rotational center). After the force is released, the residual shifts are usually small, except for the 1° yaw rotation. This test was done with the couch angle at 0°.

Table 1 shows the results of forced movement among nine volunteers using the Freedom system and measured with AlignRT. This represents the worst‐case movement that could be expected in the clinic. For all four forced movements, the average motion amplitude in each direction is about 2±1 mm, much smaller than that from open face mask with a standard head rest.^(15)^ Figure 4 shows an example of forced movement for a single patient. It must be noted that forced movement did not result in pure 1D translational movement, but was often associated with rotations and secondary translational motions. In most cases, when the subjects relaxed, the position settled back within ± 1 mm/°.

**Table 1:** Forced movement (in mm) with the Freedom system and nine volunteers, who were asked to move forcefully to the right, left, to move their chin up or down, under surveillance with AlignRT RTD (Real‐Time Delta) for motion monitoring. Motion was confirmed with the movement of a marker point on the nose in reference to the room lasers. Note: These motions may be associated with head rotations, which may make the apparent motion at the anterior ROI greater than that observed internally

	*Forced Head Motion (mm)*			
*Volunteer*	*To Left (L)*	*To Right (R)*	*Chin Up (S)*	*Chin Down (I)*
1	−1.73	0.05	−3.60	0.97
2	−3.28	3.01	−1.95	0.74
3	−2.56	1.92	−2.90	2.33
4	−2.79	3.16	−4.35	2.66
5	−1.40	3.35	−2.21	0.20
6	−2.60	2.40	−0.40	1.10
7	−2.10	1.40	−1.00	2.00
8	−2.60	2.40	−2.00	3.60
9	−2.50	3.20	−2.20	3.00
Average	−2.4	2.3	−2.3	1.9
SD	0.6	1.1	1.2	1.2

It is inconvenient and unnecessary to perform the same experiment using PinPoint system, since it would cause pain and discomfort to the teeth, suggesting very restrictive immobilization. Moreover, very little motion was evident in two‐year fSRS treatment data with PinPoint.

### B. Pre‐CBCT alignment using AlignRT

**Figure 5:**
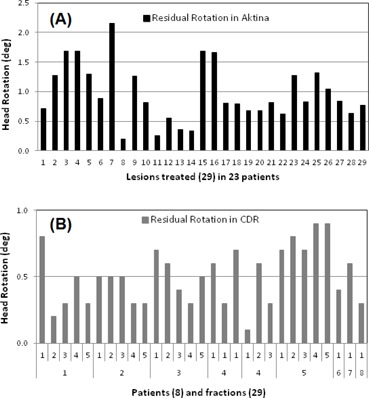
Residual head rotations (the maximum value in any three rotations) at the start of the single‐ or hypofraction SRS treatments. The PinPoint rotation adjustment board was not used (a), while Freedom head rotation adjuster (CDR) was applied (b).

Figure 5 shows the residual rotation prior to CBCT, after initial setup and rotational correction with AlignRT guidance. Manual adjustment of patient head position with PinPoint resulted in an average residual rotation (the maximum value among three rotations) of 1.1°±0.5° and an average setup time of 5.0±4.1 min, while rotational adjustment with the Freedom couch extension achieved 0.5±0.2° in about 2.7±1.0 min. The time saving is due to the use of couch adjustment and AlignRT RTD for the Freedom system vs. patient adjustment and static AlignRT imaging for the PinPoint system.

**Figure 6:**
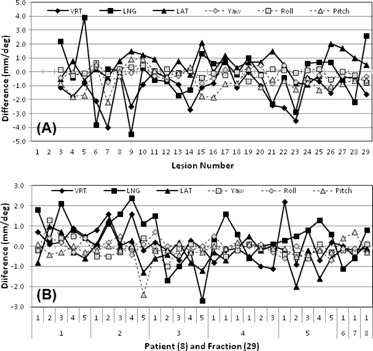
Discrepancy between AlignRT and CBCT; at CBCT registration position, residual shifts indicated by AlignRT, where the ROI and ISO are fixed: (a) PinPoint system and (b) Freedom system. The larger the residual head rotation, the greater is the observed discrepancy between CBCT and AlignRT. The residual head rotation is shown in Fig. 5.

The residual translational discrepancies between AlignRT and CBCT alignment averaged 2.4±1.3 mm for PinPoint patients and 1.5±0.7 mm for Freedom patients, as shown in Fig. 6. The discrepancy is expected to be similar for both systems, since this feature is independent of immobilization and depends primarily on imaging modality differences (AlignRT versus CBCT), their calibration, and patient deformation. The higher discrepancies (>3 mm) in PinPoint cases are due to hyperextended head positions, which lead to more uncertainty in AlignRT imaging. The residual head rotation also leads to higher residual discrepancies since the 4DOF CBCT/CT registration was applied, while the AlignRT provides 6DOF registration.

### C. Setup verification and motion monitoring during treatment

**Figure 7:**
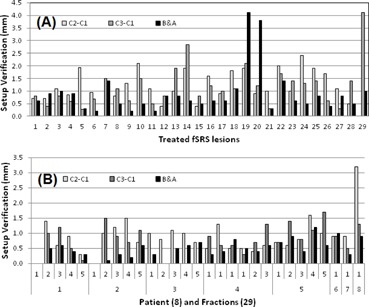
Differences between setup verification at different couch angles and initial setup using AlignRT. C2–C1 is the translational difference between second and first couch angles. C3–C1 is the shift between the third and the first couch angles. B&A is the shift difference before and after treatment. For most of the differences larger than 1.5 mm, the patient head position was hyperextended and a further verification was performed at couch zero. With PinPoint (a) there were two patients who moved due to problems with the initial mouthpiece being initially misplaced and then moving into position during treatment. The post‐treatment image was taken from verification image at the last couch after RTD and shows a >1.0 mm motion. Necessary shifts were made based on another CBCT. With Freedom (b), similar or slightly better results were obtained, due to avoidance of hyperextended head position.

At each treatment couch angle, the patient position was verified using AlignRT. Figure 7 shows the couch angle dependency due to the limited number of cameras in a treatment room. This factor and residual head rotation may result in apparent misalignments with AlignRT that are in addition to real misalignments due to possible patient motion and couch isocentric walk. Most of the large shifts (>1.5 mm) were found to be false positives, and disappeared after rotating the couch back to zero and reimaging with AlignRT. There are two PinPoint cases (Lesion 19 and 20 in Fig. 7(a)), in which actual patient motion occurred during treatment. In both cases, the motion was caused by initial misalignment of the dental mold that was ultimately detected and corrected. Volunteer data using Freedom system (not shown) are similar to patient data.

**Figure 8:**
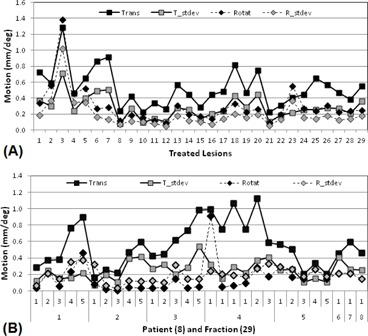
The amplitude of head motion (maximum translation and rotation values in 6DOF) during treatment, as observed with AlignRT real‐time motion monitoring: (a) PinPoint and (b) Freedom system.

Figure 8 shows the mean RTD intratreatment motion vector amplitudes and standard deviations for both translation and rotation. These data overestimate the actual motion for both systems since the beam‐off data could not be excluded and also tends to be noisy due to blockage of the AlignRT system by the gantry between beams.

### D. Assessment of patient comfort level and clinical workflow

**Figure 9:**
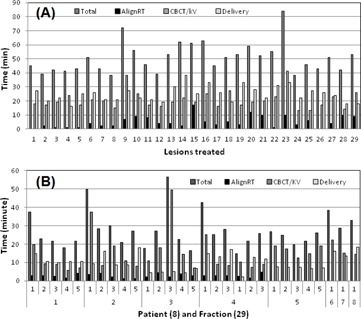
Time distributions in the fSRS procedures using the two immobilization systems: AlignRT pre‐CBCT alignment, CBCT/2D kV and treatment delivery. The averaged time for AlignRT head rotation correction is 5.0±4.0 min for PinPoint system (a) and 2.7±1.0 min for Freedom system (b). The most time‐consuming components are the CBCT acquisition, manual registration, 2D kV verification, and IGRT approval process, but the treatment delivery is relative quick.

Table 2 shows the consensus data from 15 participants (6 physicists and 9 therapists) who worked with both immobilization systems at either simulation or treatment. The mean values for patient comfort and ease‐of‐use are higher for Freedom than PinPoint. The p‐values show that the difference between the two systems is significant (p≤0.001). The treatment time for both image‐guided fSRS procedures is shown in Fig. 9.

**Table 2:** A survey of patient comfort and ease‐of‐use for each immobilization system using a scale of 1 to 10, with 1 being the lowest and 10 being the highest. Fourteen physicists and therapists with experience on both systems participated in the survey

*User*	*Physicist (P) or Therapist (T)*	*Patient Comfort*		*Ease of Use*	
		*PinPoint*	*Freedom*	*PinPoint*	*Freedom*
1	P	6	8	7	7
2	T	6	7	6	7
3	T	7	‐	8	‐
4	P	2	9	5	9
5	T	1	9	3	7
6	P	7	10	7	9
7	T	4	7	4	7
8	T	5	8	5	9
9	T	7	8	9	7
10	T	2	9	4	9
11	P	2	8	2	8
12	P	5	7	6	8
13	P	5	9	6	8
14	T	5	8	7	7
15	T	3	8	3	8
Mean		4.5	8.2	5.5	7.9
SD		2.0	0.9	2.0	0.9
p‐value		<0.001		0.001	

## IV. DISCUSSION

It is important to note that, in the fSRS treatment using both immobilization systems, a new reference AlignRT image is acquired after the patient setup is finalized based on CBCT/simCT alignment on bony landmarks. The original ROI drawn on DICOM surface from simCT is automatically mapped to the new reference image, serving as a skin surrogate of the tumor position on the treatment day. This ROI is used to offset the difference between OSI and CBCT such that the initial verification should have a perfect alignment with negligible shifts in all 6DOF. This new reference allows us to record a potential patient motion as the verification image deviates from the reference. At any nonzero treatment couch angle, a static OSI is always captured to confirm patient position, followed by motion monitoring using RTD. The head motion data of the patient can be obtained either online by capturing a new ROI at each couch angle for RTD or offline by subtracting the discrepancy in the verification at the couch angle from the RTD curve.

### A. Advantages and disadvantages of the two immobilization systems

The PinPoint system has the advantage of stringent immobilization. In both this and a previous study,^(12)^ patients were immobilized within 1.0 mm most of the time. Stringent immobilization was highly desirable during the initial transition from frame‐based to frameless SRS. Only two cases among the first 29 clinical treatments using PinPoint showed significant patient movement, as shown in Fig. 7. In both of these cases, AlignRT detected and 2D kV confirmed the movement and the patient position was corrected without any major problem.

However, the use of a highly stringent bite‐block–based immobilization system requires additional efforts to make the customized mouthpiece before the CT scan. It could also compromise the comfort of patients during treatment since it may cause pain if the mouthpiece is not exactly in the simulation position or if the locking of the mouthpiece applies some force to the patients. Furthermore, the mouthpiece could cause swallowing and breathing problems during extended period on the couch, and additionally there are about 10% of patients who simply could not tolerate treatment with the mouthpiece. For head rotation correction with PinPoint, manual head adjustment involves unlocking the arch (with which the mouthpiece is rigidly attached), repositioning the patient with the mouthpiece, and relocking the arch. This time‐consuming process can also cause discomfort, so we limit the number of attempts to three for head rotation correction. For patients with poor dentition, the setup and treatment process may lead to other kinds of discomfort, including bleeding of the gum or displacement of dental crowns.

The Freedom system offers certain advantages to the patient, including no dental visit for making the mouthpiece, ease of head mold construction at simulation, ease of rotational correction at treatment, and improved patient comfort level. However, the open face mask is less rigid. For this reason, we evaluated the magnitude of deliberate forceful movement using volunteers and found that the maximum motion is ~ 4 mm at the anterior surface, which usually translates to a smaller motion inside the cranium. This is because apparent lateral movements on the patient surface, as well as “chin‐up” or “chin down” movements, are actually the result of rotational movements of the head (Fig. 4) with the rotational center usually posterior to the midline of the head. Interestingly, when the force is released, the head returns to its original position to within 1 mm/1° most of the time.

Facial motion occurs more often in the Freedom system, because patients are more relaxed with the open face mask. Because there is no restriction on the mouth, motion in the area of the mouth during respiration may appear to be a breathing trace with amplitude of ~ 2 mm in AlignRT RTD. In this case, patients may need to be coached to try and limit this motion. In addition, the open face masks may constrict soft tissue. For patients receiving steroid treatment, facial swelling may result in changes to the anterior skin surface particularly in the area of the cheeks, leading to mask tightness and patient discomfort. Furthermore, it may make the skin ROI unreliable for initial head rotation correction using AlignRT. Because the swelling is usually not uniform within the ROI (e.g., more swelling in the cheek area than on the forehead), the change in the surface ROI could lead to inaccurate rotational alignment. If the skin swelling is observable, it is recommended to use the surface of the mask, rather than the skin surface, as the ROI for initial AlignRT rotation assessment.

In comparison to a previous study, in which the head mold alone was used as a maskless approach,^(11)^ the open face mask helps to prevent anterior and inferior motions. Therefore, if a patient falls asleep during treatment, the head remains within the tolerated position. Furthermore, since the head mold of the Freedom system has more elevation around the head than a head cushion, a greater restriction of lateral and superior motions is expected. These added stringencies in the Freedom system are expected to reduce the requirement for patient compliance and the frequency for head repositioning during treatment.

### B. Common issues in markerless AlignRT‐guided fSRS

In a previous study, we reported a direct comparison between our frameless PinPoint fSRS and frame‐based SRS.^(12)^ It showed residual patient motion with both approaches and, although the motion amplitude with PinPoint cases was slightly higher than framed cases, the study indicated that the PinPoint system with motion monitoring could serve as a suitable alternative to the conventional SRS.^(12)^ In the current study, we evaluated both the PinPoint and Freedom systems and observed that both markerless fSRS systems show similar immobilization results.

AlignRT provides real‐time optical surface imaging to monitor patient motion with high detection sensitivity without using any fiducial markers. The capability to set a motion threshold for beam‐holding via motion management interface is useful when the motion exceeds a set threshold. The nature of the nonionizing radiation of AlignRT facilitates the motion monitoring with 1) in‐room operation at patient setup, and 2) unlimited imaging throughout treatment. In the following paragraphs, a few common issues will be discussed with suggested solutions.

First, it is critical to immobilize the head in a neutral position, meaning that the virtual line from the forehead to the chin should be approximately horizontal. A hyperextended chin position makes the ceiling‐mounted AlignRT cameras view tangential facial surface, thereby causing a large uncertainty in distance determination. Such a scenario will lead to a monitoring problem or prolong treatment, due to the uncertainty that is often above the action threshold for noncoplanar beams; this is an AlignRT‐specific requirement, independent of the immobilization system.

Second, residual head rotation should be minimized, either by manually adjusting patient position initially or by adjusting head board after the patient is locked into an immobilization device. Depending on the distance from the isocenter to the anterior facial area (ROI), the effect of residual rotation on the isocenter alignment varies. This is especially important when a single isocenter is used for treating multiple lesions. In general, minimizing the residual rotation would be useful in improving treatment accuracy and reducing the discrepancy between AlignRT and CBCT.

Finally, using the facial skin surface as the intracranial tumor motion surrogate may lead to false alarms for tumor motion. Although patients can move their face in both immobilization systems, the detection of motion with the Freedom system appears more likely than with the PinPoint system, as shown in Table 1. The PinPoint mouthpiece not only immobilizes the head, but also causes mild skin stretching and is less likely to deform, while Freedom open face mask is less restrictive, allowing patients to breathe through the mouth, showing increased facial movement.

## V. CONCLUSIONS

Our comparison of two markerless fSRS immobilization systems using OSI motion monitoring provides an overview of their clinical and technical aspects. The PinPoint system provides highly stringent immobilization, resulting in minimal motion but with lower patient comfort and ease‐of‐use. The Freedom system provides a relatively less‐stringent immobilization, but has the advantage of improved patient comfort level and convenience for clinical use. Overall, based on the consensus as shown in Table 2, we recommend the Freedom system more than the PinPoint system in general; however, with OSI real‐time motion monitoring and beam‐hold capabilities when tolerances are exceeded, both markerless fSRS approaches can be applied clinically.

## ACKNOWLEDGMENTS

The authors thank the therapists at the simulation and treatment rooms for assistance in patient setup, especially Marcia Chong Ton, Gerri Pastrana, Juan Feliz, Konstantin Shuyeninov, and David Janesian, as well as all the planners for single‐fraction stereotactic radiosurgery and hypofraction stereotactic radiotherapy, including LiCheng Kuo, Cesar Della‐Biancia, Sean Berry, Sandra Fontenla, Elena Rubin, Kate Chapman, Kurt Sysock, Jingdong Li, and Jenny Guozhen Yang. The authors would also like to thank the reviewers for their valuable comments on this manuscript.
